# Fetal Growth in Pregnancies Conceived after Gastric Bypass Surgery in Relation to Surgery-to-Conception Interval: A Danish National Cohort Study

**DOI:** 10.1371/journal.pone.0090317

**Published:** 2014-03-21

**Authors:** Lone Nikoline Nørgaard, Anne Cathrine Roslev Gjerris, Ida Kirkegaard, Janne Foss Berlac, Ann Tabor, Danish Fetal Medicine Research Group

**Affiliations:** 1 Department of Obstetrics and Gynecology, Hillerød Hospital, Copenhagen University Hospital, Hillerød, Denmark; 2 Department of Obstetrics and Gynecology, Aarhus University Hospital, Aarhus, Denmark; 3 Department of Obstetrics, Copenhagen University Hospital Rigshospitalet, Copenhagen, Denmark; 4 Center of Fetal Medicine, Department of Obstetrics, Copenhagen University Hospital Rigshospitalet, Copenhagen, Denmark; 5 Faculty of Health Sciences, University of Copenhagen, Copenhagen, Denmark; The Ohio State Unversity, United States of America

## Abstract

**Objective:**

To describe early and late fetal growth in pregnancies conceived after gastric bypass surgery in relation to time from surgery to conception of pregnancy.

**Methods:**

National cohort study on 387 Danish women, who had laparoscopic or open gastric bypass surgery prior to a singleton pregnancy in which first trimester screening was performed between January 2008 and June 2011. Data were derived from national registers (Danish National Registry of Patients and Danish National Birth Registry, Pregnancy Complications and Abortion - clinical quality database (PreCAb) and the Danish Fetal Medicine Database). Main outcome measures were early and late fetal growth in relation to time from bariatric surgery to conception of the pregnancy. Early fetal growth was expressed as “Fetal Growth Index”: the ratio between the estimated number of days from first trimester ultrasound to second trimester ultrasound biometries and the actual calender time elapsed in days. Late fetal growth was expressed as the observed versus expected birthweight according to gestational age (GA).

**Results:**

The surgery-to-conception interval ranged from 3 to 1851 days with a mean value of 502 (SD, 351) days. The mean “fetal growth index” was 0.99 (SD, 0.02) days/day and thus significantly lower than in the background population (mean, 1.04 (SD, 0.09) days/day, p<0.0001).

The proportion of infants being small for gestational age was 18.8% and the proportion of large for gestational age infants was 6.7%. The correlation coefficients between surgery-to-conception time and “fetal growth index” and birthweight according to GA were 0.01 (p = 0.8) and 0.04 (p = 0.4), respectively.

**Conclusion:**

Fetal growth index was lower than reported in the background population. No correlation was found between the surgery-to-conception interval and early or late fetal growth in pregnancies conceived after gastric bypass surgery.

## Introduction

The number of bariatric surgical procedures is increasing in most of the Western countries [1]–[3]. During the years 2005–2010 the number of bariatric surgeries in Denmark increased from 279 to more than 4000 per year [1]. The most frequently used procedures are laparoscopic Roux-en-Y gastric bypass and laparoscopic gastric banding and many of these procedures are done on women of childbearing age [1]. The malabsorptive surgery (Roux-en-Y gastric bypass) results in life-long anatomic and physiologic changes and may thus cause more safety concerns in pregnancy compared to the restrictive procedures (gastric banding) [4], [5].

Among pregnant women in Denmark more than 33% are overweight or obese and because of that have increased risk of complications such as gestational diabetes, preeclampsia, cesarean delivery, macrosomia, low Apgar score and stillbirth [6]. Bariatric surgery seems to increase fertility and reduce these risks [2], [3], [7]. The primary concerns in pregnancy after bariatric surgery seem to be surgical complications to the mother (bowel obstruction and ulcers) and nutritional deficiencies for mother and infant due to malabsorption. Several reports have shown an increased risk of deficiencies in protein, iron, B12, calcium, vitamins A, D and K, which may lead to fetal growth restriction, malformations, rickets, cerebral hemorrhage, retinal damage and anemia of the fetus [2], [3], [7].

The optimal timing of pregnancy after bariatric surgery is a major concern since pregnancies conceived rapidly after surgery occur in a period of rapid weight loss which may theoretically cause fetal malnutrition. In many countries the recommendations have been to postpone pregnancy to 12–18 months after surgery when weight loss generally has reached a plateau [1], [8], [9].

The aim of our study was to evaluate fetal growth in relation to the time interval between gastric bypass surgery and conception. To do so we established a national cohort of 387 women who conceived following gastric bypass surgery.

## Methods

### Ethics statement

The study was approved by the Danish Data Protection Agency (J.nr. 2012-41-0157). Written informed consent from the women in the cohort was not required since only register data were used and ethical committee permission is not by Danish law required for register studies.

The study population is a cohort of Danish women who have had gastric bypass surgery prior to a pregnancy with a first trimester nuchal translucency scan in the period 1 January 2008 to 30 June 2011. Data were derived from the following national registers: The Danish National Registry of Patients and National Birth Registry, which are mandatory and cover all births and discharge diagnosis using the International Classifications of Diseases diagnostic codes (ICD-10) and all surgical procedures classified using a national classification system; The Pregnancy Complications and Abortion - clinical quality database (PreCAb) (http://www.tigrab.dk/) and The Danish Fetal Medicine Database, which collects data from the first trimester nuchal translucency scan and the second trimester malformation scan from all Danish Departments of Obstetrics and Gynecology. Approximately 90% of all pregnant women in Denmark participate in these ultrasound scans [10]. A personal identification number (PIN) is given at birth to every live newborn in Denmark and is used in all registrations, including health registers, thus allowing wide-ranging linkage between different national registers.

Inclusion criteria for the study were women with a Danish PIN between 15 and 49 years of age, who had a laparoscopic or open gastric bypass surgery prior to a pregnancy with a first trimester nuchal translucency scan in the period 1 January 2008 to 30 June 2011 (n = 497). Women who had undergone gastric banding were not included in the study. The outcome of the pregnancy could be either termination for any reason, spontaneous abortion or delivery of a child. Excluded from the study were women with twin pregnancies (n = 30) and women where complete information regarding ultrasound biometries from the first trimester nuchal translucency scan and the second trimester malformation scan could not be obtained (n = 80). Hence all women in the study had an ultrasound scan at 11+2 to 13+6 weeks with measurement of Crown Rump Length (CRL) and an ultrasound scan at 18+0 to 22+0 weeks when the fetus was examined for structural malformations, and biparietal diameter (BPD), occipital frontal diameter (OFD), abdominal circumference (AC) and femur length (FL) were measured. The BPD and OFD were measured in the axial plane at the level of the thalami, the third ventricle and the cavum septi pellucidi, from the outer to the outer border of the skull.

The gestational age (GA) of the fetus was estimated by means of the CRL in the first trimester (GA_12_) using the formula of Robinson and Fleming [11] and by the BPD and OFD in the second trimester (GA_20_) using the formula of Altman and Chitty [12]. The calendar time in days between the two ultrasound examinations was noted (Days _calendar_) and the fetal growth between the first and second trimester was calculated as the ratio between the estimated number of days from the first to the second scan and the actual calendar time elapsed in days (GA_20_-GA_12_)/Days_calendar_ as described by Kirkegaard et al [13]. In this way the observed versus the expected increase in fetal size was calculated, expressed as a time interval (days/day) and designated “fetal growth index”. A value of one corresponds to no deviation in growth, a value below one indicates some degree of growth restriction and a value above one indicates growth faster than expected. The “fetal growth index” was compared to a normal population of 9450 pregnancies described by Kirkegaard et al [13] and was furthermore related to the time interval from bariatric surgery to conception of pregnancy (the surgery-to-conception interval). Conception of the pregnancy was calculated as the expected date of delivery estimated from the CRL at the nuchal translucency scan minus 266 days. Scandinavian gender-specific growth curves [14] were used to evaluate small-for-gestational age (SGA) as <10^th^ percentile and large-for-gestational age (LGA) >90^th^ percentile.

All data regarding variables from the ultrasound scans were derived from the Danish Fetal Medicine Database, which was founded in 2008. Data was transferred to the database from 19 departments of Obstetrics and Gynecology in Denmark using the Astraia database (www.astraia.com) and sonographers certified by The Fetal Medicine Foundation performed all examinations.

Background data and fetal outcome were obtained from the Danish Fetal Medicine Database and the Danish National Registry of Patients and included information about maternal age, Body Mass Index (BMI) (kg/m^2^) at the first trimester scan, parity, smoking, ethnicity, method of conception, GA at delivery and birthweight of the infant. The background data were compared to a control group of all Danish pregnancies with data in the Danish Fetal Medicine Database who had a nuchal translucency scan in 2008 (n = 57,368).

Student's *t*-tests were used to compare mean values between continuous normally distributed data and proportions between groups on a dichotomous response were compared with chi-squared analysis using Yates' correction. Linear regression (Pearson) analyses were used for calculations of fetal growth according to surgery-to-conception interval. Excel 2007 and Graph Pad Software were used for analyses.

## Results

A total number of 387 women who had a pregnancy after gastric bypass surgery met the inclusion criteria and were included in the study. Maternal characteristics and lifestyle factors are presented in Table 1. Women in the study group had higher BMI than the general population of pregnant women (range 18–57 kg/m^2^). One woman (0.3%) was underweight with BMI <18.5 kg/m^2^; 34 (9.0%) were in the normal range with BMI 18.5–24.9 kg/m^2^; 134 (35.4%) were overweight with BMI 25–29.9 kg/m^2^ and 210 (55.4%) were obese with BMI ≥30 kg/m^2^. Data on age, BMI, assisted reproduction and smoking were almost complete with missing values in less than 2.5% in the study group and less than 10% in the general population of pregnant women (Table 1).

**Table 1:** Maternal characteristics of women with gastric bypass prior to their pregnancy compared to all pregnancies registered in the Danish Fetal Medicine Database in 2008.

Characteristics	Gastric bypass (n = 387)	All (n = 57,368)	P value^a^
Age (mean, years)	30.8 (SD, 4.8)	29.9 (SD, 4.9)	<0.001
Smoking^b^	65/383 (17.0%)	6225/56,691(11.5%)	<0.001
BMI^c^ (mean, kg/m^2)^)	30.7 (SD, 5.4)	23.9 (SD, 5.0)	<0.001
Assisted reproduction^d^	29/378 (7.7%)	3670/52,154 (7.0%)	NS^e^
Parity (mean)	1.04 (SD, 1.0)	0.99 (SD, 1.1)	NS^e^

a *Continuous normally distributed data were compared with Student's t-test and proportions between dichotomous outcomes were compared with χ^2^-square tests using two-sided p-values.*

b *Data on Smoking were missing in 4/387 women in the gastric bypass group and 677/57,368 in the general pregnant population.*

c *Body mass index. Missing values on BMI in 8/387.*

d *ICSI, IVF and insemination, missing information on 9/387 in the gastric by pass group and 5214/57,368 in the general pregnant population.*

e *not significant.*

The surgery-to-conception interval ranged from 3 to 1851 days with a mean value of 502 (SD, 351) days. The woman with a calculated surgery-to-conception interval of 3 days was 21 year old with a BMI of 34 kg/m^2^, expecting her second child. She delivered 13 days prior to the expected date of delivery and although gestational age was based on a first trimester ultrasound scan with measurement of CRL, the pregnancy may have been conceived prior to surgery.

The “fetal growth index” ranged from 0.93 days/day to 1.07 days/day among all fetuses and the mean index was 0.99 (SD, 0.02) days/day and thus significantly lower than reported by Kirkegaard et al in a population of 9450 singleton normal pregnancies (mean, 1.04 (SD, 0.09) days/day, p<0.0001) [13].

**Figure 1:**
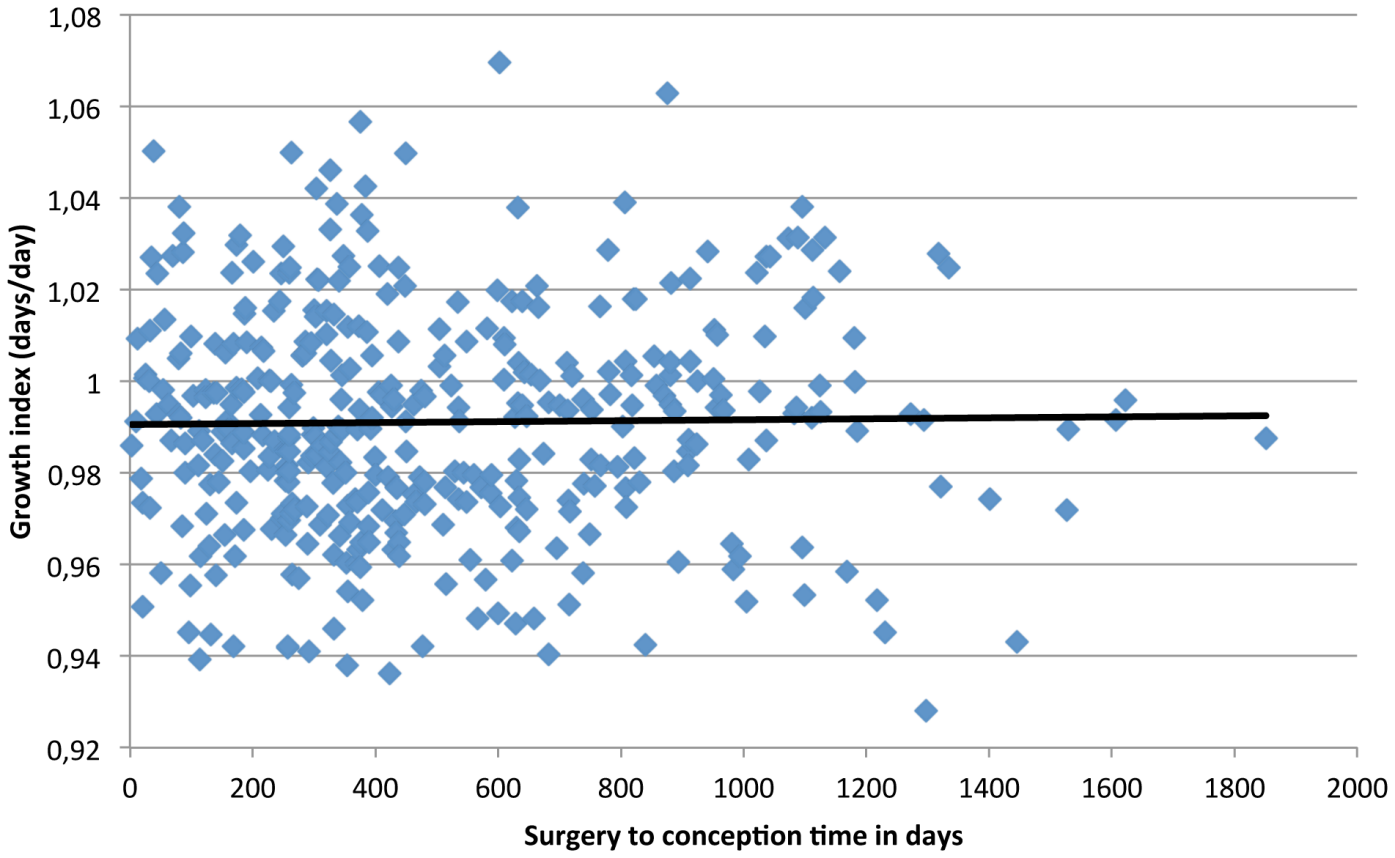
Relation between surgery-to-conception time and fetal growth index.

**Figure 2:**
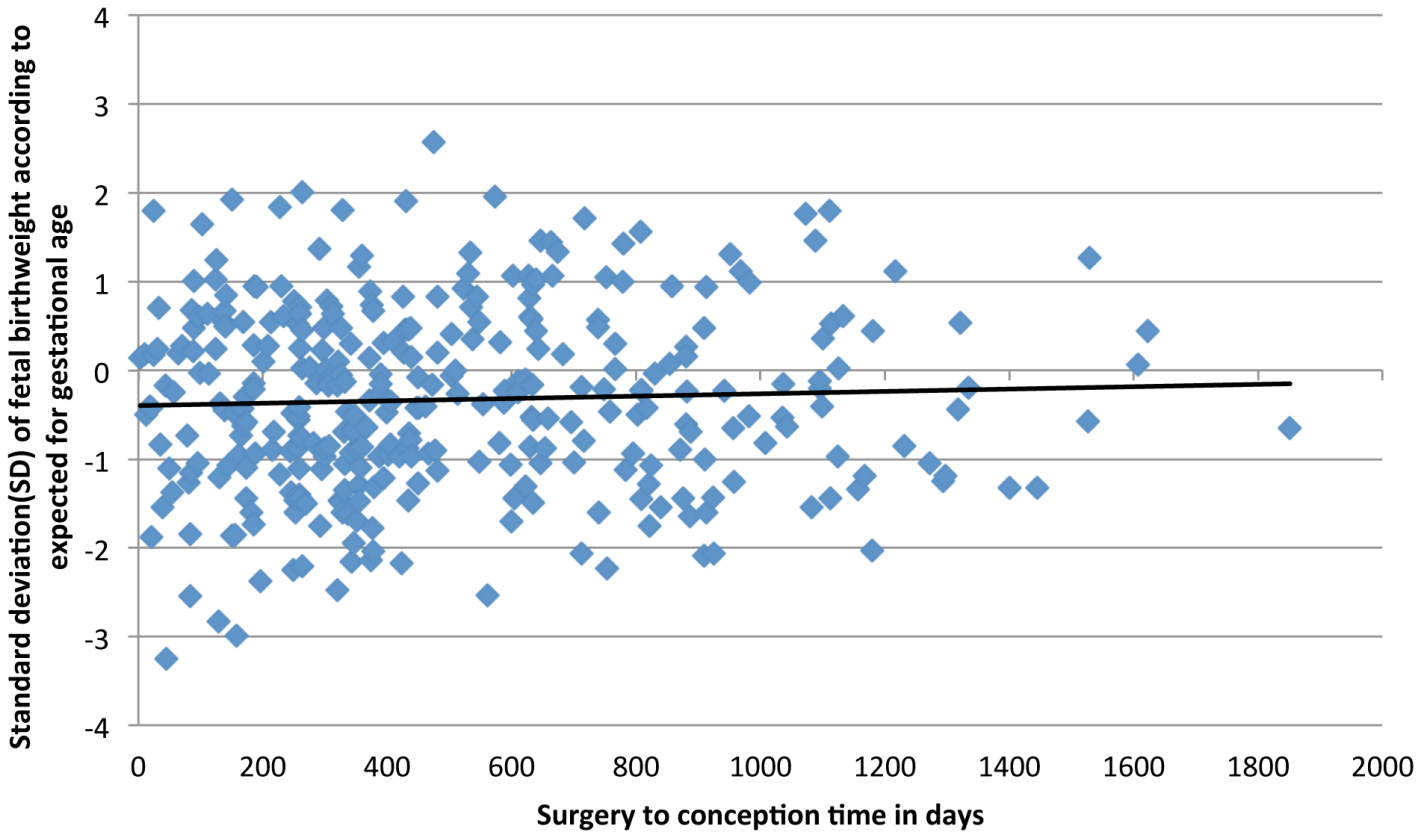
Relation between surgery-to-conception time and fetal birthweight according to expected for gestational age.

The relation between surgery-to-conception interval and fetal growth is shown in Figure 1 (relation to “fetal growth index”) and Figure 2 (relation to SD of the expected versus the observed birthweight according to GA). The correlation coefficients were 0.01(p = 0.8) and 0.04 (p = 0.4), respectively, and thus this study shows very weak if any relation between time from surgery to conception and early fetal growth or risk of the infant being SGA. There was no statistically significant difference in SGA in pregnancies conceived before or after 18 months from surgery (19.7% versus 18.0%, p = 0.8).

Gestational age at the nuchal translucency scan, at malformation scan, GA at delivery and birthweight of the infant are presented in Table 2. Women with gastric bypass surgery had significantly higher GA at the malformation scan than women in the general population. The unadjusted GA at delivery and birthweight were significantly lower in the bariatric surgery group compared to women in the general population. Data on birth weight and GA were available in 336/387 cases. Calculations on SGA and LGA are based on these 336 pregnancies.

**Table 2:** Pregnancy characteristics and outcome in women with gastric bypass prior to their pregnancy compared to all pregnancies registered in the Danish Fetal Medicine Database in 2008.

Characteristics	Gastric bypass (n = 387)	All (n = 57,368)	P value^a^
GA at NT scan (mean, days)	88.8 (SD, 4.2)	89.3 (SD, 5.9)	0.11
GA at malformation scan (mean, days)	141.3 (SD, 9.5)	139.3 (SD, 7.8)	<0.001
GA at delivery (mean, days)	273.2 (SD, 15.8)^b^	277.8 (SD, 14.0)	<0.001
Birth weight (mean, gram)	3271 (SD, 580)^c^	3491 (SD, 589)	<0.001

*GA = Gestational age, NT = Nuchal Translucency.*

a *Continuous normally distributed data were compared with Student's t-test, unpaired using two-sided p-values.*

b *missing values on GA in 46/387.*

c *missing values on birth weight in 51/387.*

The overall proportion of newborns being SGA in the study group was 64/336 (19.0%) and the proportion of LGA newborns was 22/336 (6.5%). The proportion of SGA newborns in women with BMI less than 25 was 8/27 (29.6%), 23/117 (19.6%) in women with BMI 25–30 and 31/185 (16.8%) in women with BMI above 30, showing no significant difference (p = 0.3). The risk of LGA was 0/27 (0%), 5/117 (4.3%) and 17/185 (9.2%) in women with BMI below 25, between 25 and 30 and above 30, respectively, (p = 0.09). Only one woman in the study group (BMI 40 kg/m2) had a newborn with macrosomia (birthweight >4500 g). The proportions of SGA newborns with GA below 37 weeks was 7.8% compared to 4.5% of LGA newborns and 9.2% of the newborns with birth weight appropriate for GA (no significant difference).

## Discussion

In this national cohort study on 387 pregnancies conceived after gastric bypass surgery we found no relation between early fetal growth and the surgery-to-conception interval. Nor did we find any relation between birthweight according to GA and the time from surgery to conception. The current recommendations of delaying pregnancy until after 12–18 months [1], [8], [9] are based on theoretical risks. Speculation has been that the catabolic phase and dramatic weight loss the first year after surgery as well as vitamin deficiencies could have severe impact on fetal growth. Many national guidelines have drawn attention to this issue, recommending close monitoring with assessment and supplementation of folate, iron, calcium and vitamin A, D, K and B12 [1], [8] and it is possible that the benefits of these recommendations are seen in our study population. The first Danish national guideline on antenatal surveillance of pregnancies in women with prior gastric bypass was accepted in 2012 (www.dsog.dk) recommending extensive obstetrical surveillance in dedicated units with ultrasound for fetal weight, blood sampling for vitamin deficiencies, home blood glucose monitoring etc. This means that during the study period 2008–2011 these women have been followed by local protocols. The Danish guideline on screening for gestational diabetes from 2002 (www.dsog.dk) suggest screening of women with BMI ≥27 kg/m^2^, family history of diabetes or prior child with birth weight above 4500 g. Most of the women in our study will due to these criteria have been offered screening for gestational diabetes.

A French multicenter study on 94 pregnancies after gastric bypass surgery of the mother (63 with gastric banding and 31 with Roux-en-Y gastric bypass) showed no significant difference in birthweight or SGA in pregnancies conceived before or after one year of surgery [4]. Another reported series of 34 patients with pregnancy after gastric bypass surgery showed no difference in birthweight between the 21 patients who became pregnant within the first year after surgery compared to the 13 women with conception after one year after surgery [15]. Our study support these and other small series that show similar neonatal outcomes in women with pregnancies conceived before or after the period of maximal weight loss [16]–[18].

One of the major complications in pregnancies in obese women is the risk of macrosomia. In a Danish study on 403,092 Danish women giving birth between 2004 to 2010 Ovesen et al reported 5,589 cases of macrosomia in 120,411 women with BMI above 25 kg/m^2^ (4.6%) [6]. In our study population we had 344 women with BMI above 25 kg/m^2^ and only one case of macrosomia (0.003%). This is statistically less than reported by Ovesen (p = 0.0002) supporting the findings of others – that bariatric bypass surgery decreases the risk of macrosomia compared to BMI matched controls [5], [16], [19], [20]. In our study there was a tendency towards higher proportion of LGA with increasing BMI, but the difference was not statistically significant due to the small numbers in the subgroups.

Increased risk of SGA in pregnancies after gastric bypass has been reported. Lesko and Peaceman [19] report 17.4% of infants being SGA in a group of 70 women with pregnancy after gastric bypass surgery compared to 5.0% among 140 BMI matched controls (p<0.01). Another study of 162 pregnancies after gastric surgery with biliopancreatic diversion compared to pregnancies prior to surgery, also reported a higher proportion of SGA newborns (9.6% versus 3.1%, p<0.05) [20] and the same tendency was reported by others [4], [16], [18]. We found an overall SGA proportion of 18.8% with a tendency to decreasing risk with increasing BMI. Further we found the mean early fetal growth index to be significantly lower than the mean early fetal growth index in 9450 Danish singleton pregnancies reported by Kirkegaard [13]. The study group of women with gastric bypass surgery may be different from the general population in many ways and a limitation of this study was the lacking ability to adjust for socio-demographic status due to non-available data, which could very well influence the “fetal growth index” and the risk of SGA and LGA due to nutritional status, smoking and exercise. A Scandinavian study has recently shown that women who have had bariatric surgery are more likely to have parents of lower socio-demographic status [21]. The significantly higher proportion of smokers among the women with bariatric surgery in our study compared to the general population in Denmark supports this (Table 1). We chose to include only women with gastric bypass surgery and exclude women with gastric banding. Gastric bypass surgery is a procedure resulting in life-long anatomic and physiologic changes including malabsorption and is therefore thought to have bigger impact on pregnancy outcomes [4], [5]. This should be taken into consideration when comparing to the findings of others since different methods of bariatric surgery (gastric banding, biliopancreatic diversion and Roux-en Y gastric bypass) are often included. The strength of register studies is the large number of individuals and the absence of selection bias. Limitations include the lacking ability to adjust for variables not in the database. In our study information on socio-demographic status, gestational weight gain , diabetes and detailed information on offspring morbidity is not available. Gestational weight gain might be an important determinant of fetal growth and could also be influenced by the surgery-to-conception interval. Controlling for gestational weight gain in the correlations to fetal growth would be optimal but unfortunately not feasible within the limits of our dataset.

In conclusion this national cohort study supports previous findings of lower birthweight and increased risk of SGA, but decreased risk of LGA in pregnancies conceived after gastric bypass surgery. Early “fetal growth index” was lower than reported in the background population, but no significant relation was found between the surgery-to-conception interval and early fetal growth or risk of SGA. So while surgery-to-conception interval may not be related to SGA, an increased risk of reduced fetal growth still remains and more studies are needed to provide information on maternal safety and neonatal outcomes in pregnancies conceived after bariatric surgery.
